# Optimization of technology for dietary fiber extraction from Maixiansan by response surface methodology

**DOI:** 10.1186/1749-8546-7-28

**Published:** 2012-12-29

**Authors:** Hui-qing Lv, Changfeng Hu, Hai-ping Zhong, Hong-bin Zheng, Chengping Wen

**Affiliations:** 1 College of Pharmaceutical Sciences, Zhejiang Chinese Medical University, Hangzhou, 310053, People’s Republic of China; 2 College of Basic Medical Sciences, Zhejiang Chinese Medical University, Hangzhou, 310053, People’s Republic of China

## Abstract

**Background:**

Our study aims to determine whether response surface methodology can optimize the extraction of dietary fiber from Maixiansan.

**Methods:**

A Box–Behnken design was employed to optimize the extraction parameters, including α-amylase concentration (X_1_: 0.3 – 0.5%), enzymolysis time (*X*_2_: 30 – 60 min) and NaOH content (X_3_: 1.0 – 5.0%), of dietary fiber from Maixiansan using an enzyme–alkali extraction technique.

**Results:**

The optimal technological conditions were as follows: α-amylase concentration: 0.4%; enzymolysis time: 45 min; NaOH content: 4.0%. Under these conditions, the extraction yield reached 57.14%, which was well consistent with the predicted models with a coefficient of determination (R^2^) of 0.9818. An evaluation of the anti-inflammatory activity indicated that Maixiansan was able to significantly inhibit dextran sodium sulfate-induced ulcerative colitis in rats by increasing the concentration of short-chain fatty acids (acetate, propionate and butyrate), among which the butyrate content was significantly higher in the Maixiansan group than in the other groups.

**Conclusion:**

Our experiments showed that response surface methodology can optimize the extraction of dietary fiber from Maixiansan. Maixiansan could be explored as an anti-ulcerative colitis agent.

## Background

Inflammatory bowel disease includes Crohn’s disease and ulcerative colitis (UC). UC is a chronic inflammatory disorder of the colon characterized by alternating periods of flare-ups and quiescent disease [1]. Dietary fiber is an integral constituent of normal human nutrition and may contribute to a healthy colonic environment [2]. The protective effects of a fiber-rich diet have been attributed to short-chain fatty acids (SCFAs), including acetate, propionate and butyrate [3]. SCFAs are the major end products of fiber fermentation in the gastrointestinal tract [4-6]. Our previous study revealed that germinated barley foodstuff (GBF) increased the numbers of eubacteria and bifidobacteria in experimental rats. Maixiansan, composed of GBF, tuckahoe and Chinese yam, can effectively prevent bloody diarrhea and mucosal damage, and exerts significant effects on UC [7].

The common approach of an orthogonal test design is usually adopted to optimize the extraction conditions for dietary fiber from Maixiansan (DFM) to achieve higher yield and quality, but the effects of interactions between different factors remain unclear. To optimize the processing parameters, response surface methodology (RSM), which is an effective statistical technique for optimizing complex processes, has recently been used to allow more efficient and easier arrangement and interpretation of experiments [8-11], by reducing the number of experimental trials needed to evaluate multiple parameters and their interactions [12-14].

This study aims to optimize the extraction parameters (α-amylase concentration, enzymolysis time and NaOH content) of DFM agents using a three-level, three-variable Box–Behnken design (BBD). The BBD is a type of response surface design. It is an independent quadratic design, in that it does not contain an embedded factorial or fractional factorial design [15].

## Materials and methods

### Materials

#### Reagents and apparatus

Maixiansan was prepared by mixing GBF, tuckahoe and Chinese yams (Rhizoma dioscoreae and Dioscorea opposita, from Henan Province, China) in certain proportions (4:2:1:1), followed by grinding with a grinder and passage through a 400-mesh sieve. All of the chemicals used were of reagent grade. Acetic acid (≥ 99.5%), propionic acid (≥ 99.5%) and butyric acid (≥ 99.0%) were purchased from Sinopharm Chemical Reagent Co. Ltd. (China). The testing tool was a GC-2010AFAPC gas chromatographic analyzer (Shimadzu, Japan) with an FID detector (Shimadzu, Japan) and an AOC-20i liquid autosampler (Shimadzu, Japan). The analytical column was a DB-FFAP capillary column (30 m × 0.25 mm i.d.; Agilent, USA). A scientific automatic microplate reader (Multiskan Spectrum) was purchased from Thermo Fisher (USA).

### Extraction of DFM

Maixiansan (2 g) was soaked in boiled water and hydrolyzed by α-amylase (Sciencelab, China) until the enzyme was inactive. The treated sample was washed with water and separated by centrifugation at 10,000 × *g* for 5 min (Eppendorf 5430R: Germany). The residue was dried in a rotary evaporator (RE52A; Shanghai Yarong Biochemistry Instrument Factory, China) to yield dietary fiber. The percentage of dietary fiber (%) was calculated by the weight of the residue.

### Optimization of DFM extraction

A BBD with three independent variables was used for the optimization. The α-amylase concentration (X_1_), enzymolysis time (*X*_2_) and NaOH content (X_3_) were the independent variables selected to optimize the extraction of dietary fiber. The ranges of the independent variables and their levels are presented in Table 1. Each variable had three levels of −1, 0 and 1 representing low, middle and high levels, respectively. The extraction yield of DFM (Y) was taken as the response for the combinations of the independent variables shown in Table 2. The experimental runs were randomized to minimize the effects of unexpected variability in the observed responses.

**Table 1:** BBD and yields of DFM

**Levels**	**α-Amylase concentration X**_**1**_**(%)**	**Enzymolysis time *X***_**2**_**(min)**	**NaOH content X**_**3**_**(%)**
−1	0.3	30	1.0
0	0.4	45	3.0
1	0.5	60	5.0

**Table 2:** RSM and results of the BBD with three independent variables

**Run**	**α-Amylase concentration X**_**1**_**(%)**	**Enzymolysis time *X***_**2**_**(min)**	**NaOH content X**_**3**_**(%)**	**Yield (%)**
1	0.3	45.00	1.0	54.73
2	0.3	45.00	5.0	56.33
3	0.3	30.00	3.0	55.51
4	0.4	45.00	3.0	56.76
5	0.4	60.00	5.0	55.8
6	0.4	45.00	3.0	56.47
7	0.3	60.00	3.0	55.85
8	0.5	30.00	3.0	55.41
9	0.5	45.00	1.0	54.56
10	0.4	30.00	5.0	55.69
11	0.4	60.00	1.0	54.29
12	0.4	45.00	3.0	56.66
13	0.5	45.00	5.0	56.09
14	0.5	60.00	3.0	55.57
15	0.4	30.00	1.0	54.1

The variables were coded according to the following equation:

(1) $$X_{i}=\frac{X_{i}-X_{0}}{\mathrm{\Delta X}}$$

where X_i_ is the (dimensionless) coded value of the variable X_i_, X_0_ is the value of X_i_ at the center point and ΔX is the step change.

The behavior of the system was explained by the following quadratic equation:

(2) $$Y=\beta_{0}+\sum_{i=1}^{i=3}\beta_{i}X_{i}+\sum_{i=1}^{3}\beta_{\text{ii}}X_{i}^{2}+\sum_{i=1}^{2}\sum_{j=i+1}^{3}\beta_{\text{ij}}X_{i}Y_{j}$$

where Y is the estimated response, *β*_*0*_, *β*_*i*_, *β*_*ii*_ and *β*_*ij*_ are the regression coefficients for the intercept, linearity, square and interaction, respectively, and X_i_ and X_j_ are the independent variables.

### Animals and diets

Forty male Sprague–Dawley rats (145 – 155 g) were purchased from the Center Animal House of Zhejiang Chinese Medical University (China), and randomly divided into four groups of 10 rats as follows: normal group; model group; mesalazine group; and Maixiansan group. The rats were acclimatized to the experimental facility for 1 week and maintained in cages in a specific pathogen-free environment in an animal facility under standard conditions (50 – 60% humidity, 12-h/12-h light/dark cycle).

UC was induced by adding 3% dextran sodium sulfate (DSS) (Pharmacia, USA) to the drinking water for 7 days. The normal group was administered clean water and the other three groups were administered 3% DSS solution. The solution was prepared every day. After 7 days, the Maixiansan group was fed 10% Maixiansan medicinal powder, while the other groups were fed the normal diet (rat diet, complying with GB 14924.3-2001). The rats in the mesalazine group were administered mesalazine for 2 weeks by gastric perfusion. All the procedures were performed according to the Animal Care Committee for the Use of Experimental Animals at Zhejiang Chinese Medical University (China).

### Fecal SCFA concentrations

Fresh morning rat stools were collected, weighed and stored at 4°C. Approximately 2 g of homogenized stool was mixed with 6 mL of sterilized water. After centrifugation at 10,000 × *g* for 20 min, the filtered supernatant was collected and the major SCFAs i.e., acetate, propionate and butyrate, were measured by capillary gas chromatography as previously described [16,17].

### Statistical analysis

All of the data were represented as the mean ± standard deviation (SD) of three replicate determinations, with a 1significance level of *P* < 0.05 for analysis of variance (ANOVA) and processing with SPSS 15.0 software (Cabit, China). Design Expert software trial version 7.0.0 (Stat-Ease, USA) was employed for the regression analysis and graphical optimization.

## Results and discussion

### Optimization of extraction conditions by the BBD

#### Statistical analysis and model fitting

The current orthogonal test design in the extraction of polysaccharides mainly focuses on arranging reasonable experiments that can deal with several factors simultaneously and find optimal factor levels, but cannot give a regression equation for the whole parameter space tested. Response surface optimization establishes a high precision regression equation, and details the interactions between several factors, in a highly efficient time-saving design pattern [15,18]. The values for the independent process variables (X_1_, *X*_2_ and X_3_) measured and the predicted values for the extraction yields of DFM are shown in Table 2. The yields ranged from 54.1% to 56.76%. The maximum yield of DFM was reached with α-amylase concentration of 0.4%, enzymolysis time of 45 min and NaOH content of 3%. By applying a multiple regression analysis to the experimental data, the response variable and the test variables can be related by the following polynomial equation:

(3) $$Y=56.63-0.0{99x}_{1}+0.100x_{2}+0.{78x}_{3}-0.0{45x}_{1}x_{2}-0.0{18x}_{1}x_{3}-0.020x_{2}x_{3}-0.{{29x}_{1}}^{2}-0.{{75x}_{2}}^{2}-0.{{91x}_{3}}^{2}$$

where Y is the extraction yield of DFM, ansd X_1_, *X*_2_ and X_3_ are the values for the α-amylase concentration, enzymolysis time and NaOH content, respectively.

The determination coefficient (R^2^ = 0.9818) was evaluated by ANOVA of the quadratic regression model. The adjusted determination coefficient (R^2^ = 0.9863) also suggested that the model was highly significant. At the same time, the low value (0.18) of the variation coefficient suggested a very high degree of precision and good reliability of the experimental values.

*P* values were used to determine the significance of each coefficient, which indicated the pattern of interactions between the variables. Smaller *P* values indicated increasing significance for the corresponding coefficients. It can be concluded that the linear coefficients (X_1_, *X*_2_ and X_3_) and quadratic term coefficients (X_1_^2^, X_2_^2^ and X_3_^2^) were significant, with small *P* values (*P* < 0.05) as shown in Table 3 [for example X_1_, *P* = 0.0366; *X*_2_, *P* = 0.0351, etc.]. The coefficients for the other terms were not significant (X_1_*X_2_, *P* = 0.4033; X_1_*X_3_, *P* = 0.7371; *X*_2_*X_3_, *P* = 0.7018). The full model according to equation (3) was shown in three-dimensional and contour plots to reveal the relationships between the independent and dependent variables. The lack-of-fit F-value (0.08) was not significant relative to the pure error.

**Table 3:** Regression coefficient estimates for the quadratic polynomial model

**Source**	**Degrees of freedom**	**Sum of squares**	**Mean square**	**F value**	***P *value**
Model	9	9.91	1.10	113.21	< 0.0001
X_1_	1	0.078	0.078	8.02	0.0366
*X*_2_	1	0.080	0.080	8.23	0.0351
X_3_	1	4.85	4.85	498.88	< 0.0001
X_1_^2^	1	0.32	0.32	32.76	0.0023
X_1_*X_2_	1	0.0081	0.0081	0.83	0.4033
X_1_*X_3_	1	0.0012	0.0012	0.13	0.7371
X_2_^2^	1	2.08	2.08	214.28	< 0.0001
*X*_2_*X_3_	1	0.0016	0.0016	0.16	0.7018
X_3_^2^	1	3.05	3.05	313.54	< 0.0001
Lack of fit	3	0.005225	0.001742	0.08	0.9648

The results also showed that NaOH content was the most significant impact factor for the extraction yield of DFM based on the smallest *P* value (X_3_, *P* < 0.0001), while those of the two other parameters were > 0.01 (X_1_, *P* = 0.0366; *X*_2_, *P* = 0.0351).

#### Optimization of the procedure

**Figure 1:**
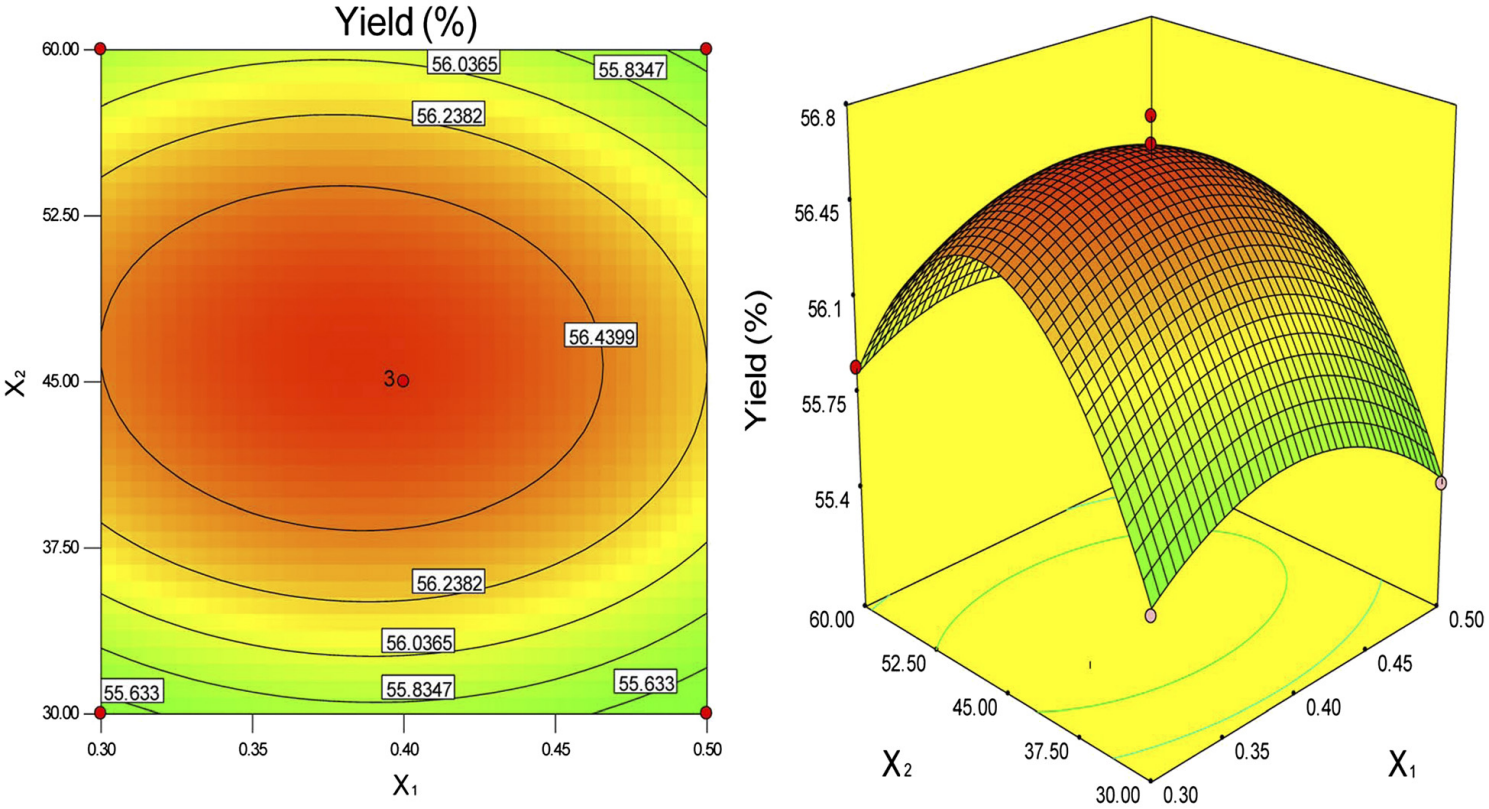
**Contour plot and response surface plot of Y = f1 (X**_**1**_**, *X***_**2**_**) (α-amylase concentration and enzymolysis time are variable; NaOH content is fixed).**

**Figure 2:**
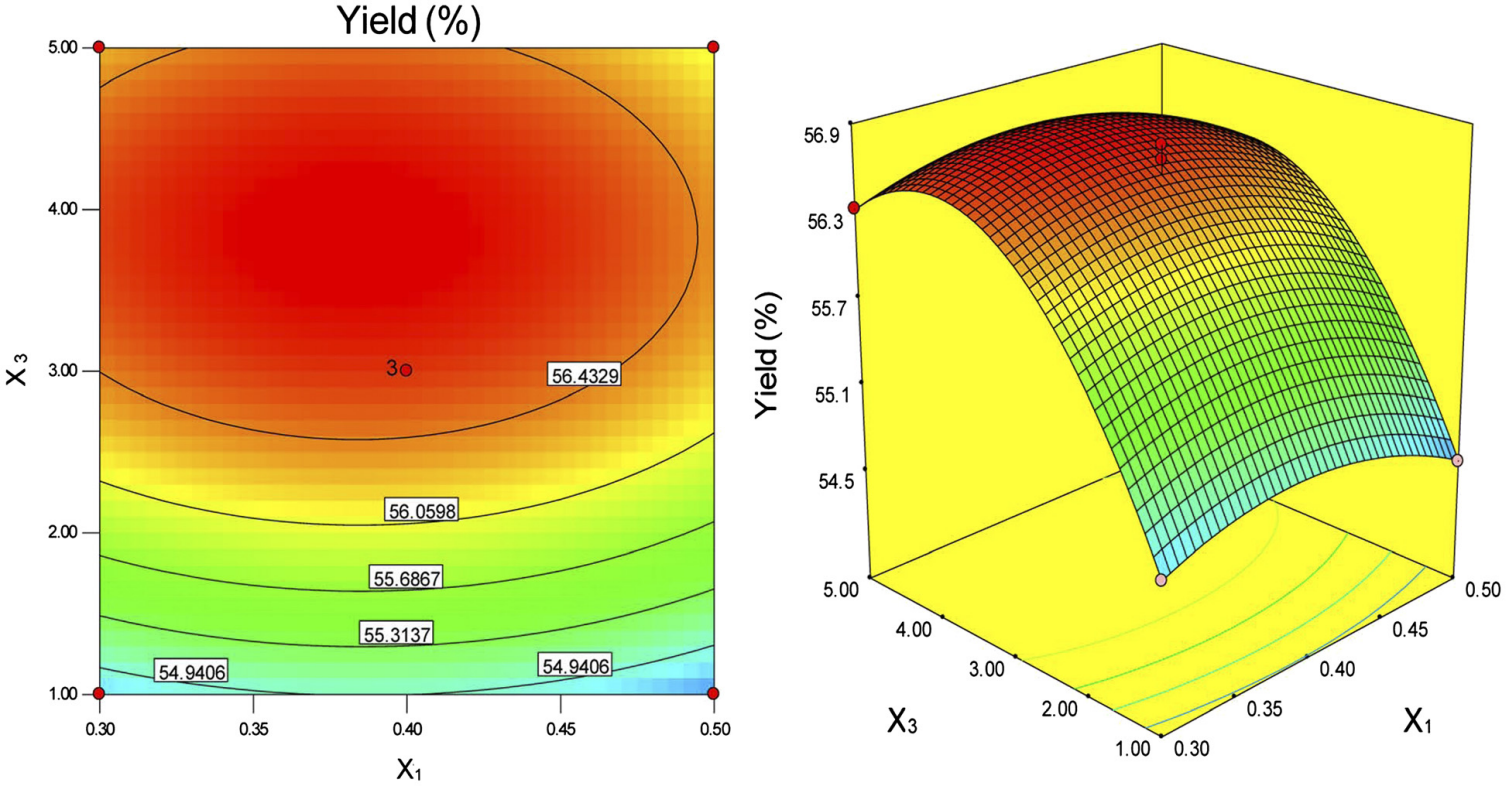
**Contour plot and response surface plot of Y = f2 (X**_**1**_**, X**_**3**_**) (α-amylase concentration and NaOH content are variable; enzymolysis time is fixed).**

**Figure 3:**
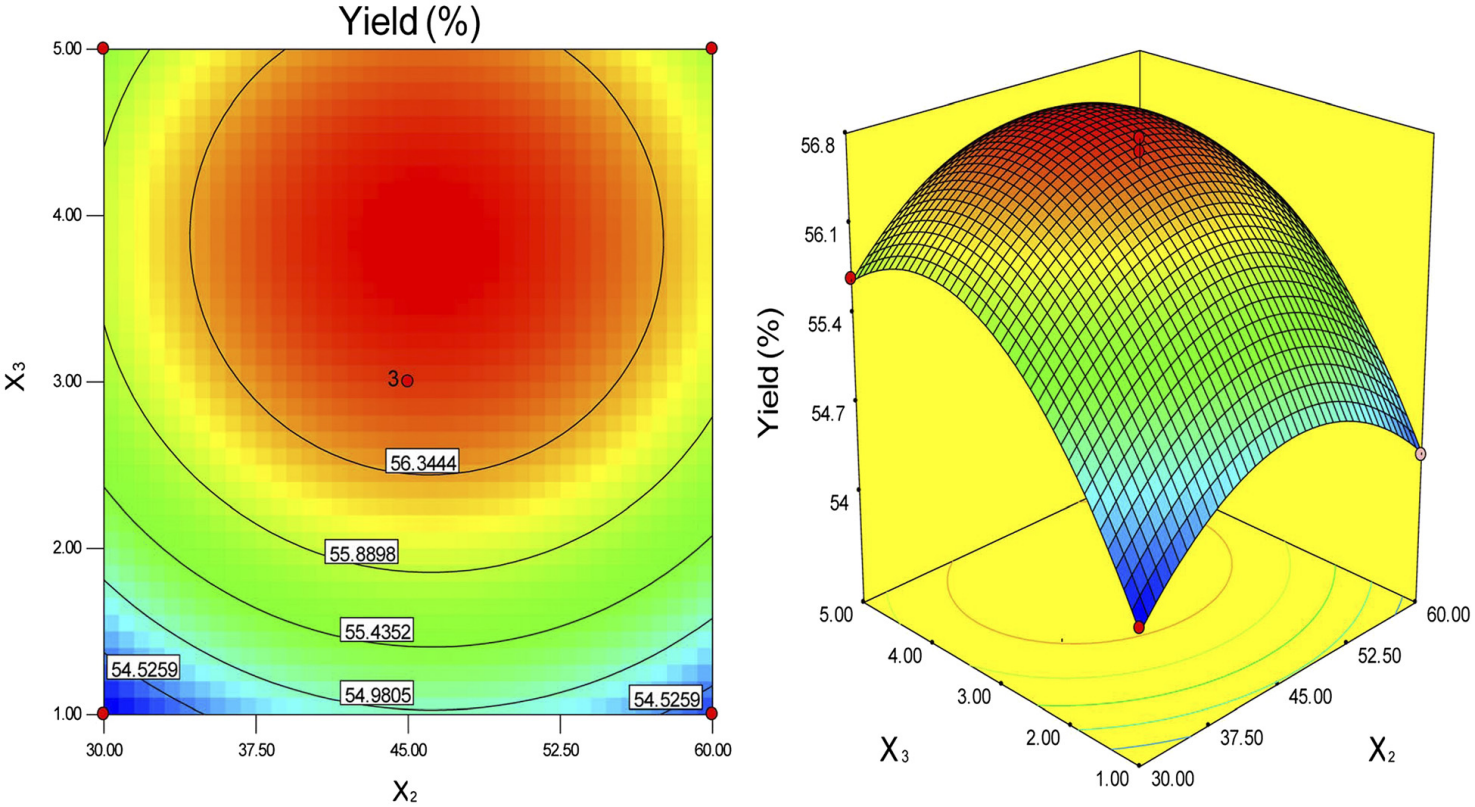
**Contour plot and response surface plot of Y = f3 (*X***_**2**_**, X**_**3**_**) (enzymolysis time and NaOH content are variable; α-amylase concentration is fixed).**

The response surfaces were plotted by Design Expert software to examine the effects of the variables and their interactions on the extraction yields of DFM. The relationships between the independent and dependent variables were illustrated by two-dimensional contour plots generated by the model and three-dimensional response surface plots (Figures 1, 2 and 3). These plots showed the effects of two factors on the response at a time when the third variable was fixed (0 level). The shapes of the contour plots, elliptical or circular, indicated whether or not the interactions between the corresponding variables were significant [19]. An elliptical contour plot meant that the interactions between the variables were significant, while a circular contour plot meant the opposite.

Figure 1 shows that the extraction yield of DFM increased with increasing α-amylase concentration and enzymolysis time from 30 to 45.99 min. However, beyond 45.99 min, the yield of DFM decreased with increasing enzymolysis time. Figure 2 shows that the maximum yields of DFM were achieved when the α-amylase concentration and NaOH content were 0.38% and 3.86%, respectively. Figure 3 shows that the extraction yields of DFM clearly increased with increasing NaOH content from 1% to 3.86%, but decreased when the NaOH content exceeded 3.86%. No significant further improvements and interactions between the variables were observed. Among the three parameters, NaOH content was the most significant factor affecting the extraction yield of DFM, followed by enzymolysis time and α-amylase concentration according to the significance of the regression coefficients of the quadratic polynomial model (Table 3) and the gradients of the slopes in the three-dimensional response surface plots (Figures 1, 2 and 3).

According to Figures 1, 2 and 3, the optimal values of the tested variables for a DFM yield of 56.81% can be predicted as follows: α-amylase concentration of 0.38%; enzymolysis time of 45.99 min; and NaOH content of 3.86%. However, considering the operability for actual production, the optimal conditions can be modified as follows: α-amylase concentration of 0.4%; enzymolysis time of 45 min; and NaOH content of 4%.

To validate the adequacy of the model equations, we carried out a verification experiment according to the optimal conditions mentioned above. Under these conditions (Table 4), the experimental yield from real experiments was 57.14% (n = 3), and consistent with the predicted value of 56.81%.

**Table 4:** Predicted and experimental values of the responses under the optimum and modified conditions

	**α-Amylase concentration (%)**	**Enzymolysis time (min)**	**NaOH content (%)**	**Yield (%)**
Optimum conditions	0.38	45.99	3.86	56.81 (predicted)
Modified conditions	0.4	45	4	57.14 ± 0.052% (actual)

#### Comparison of fecal SCFA concentrations

**Figure 4:**
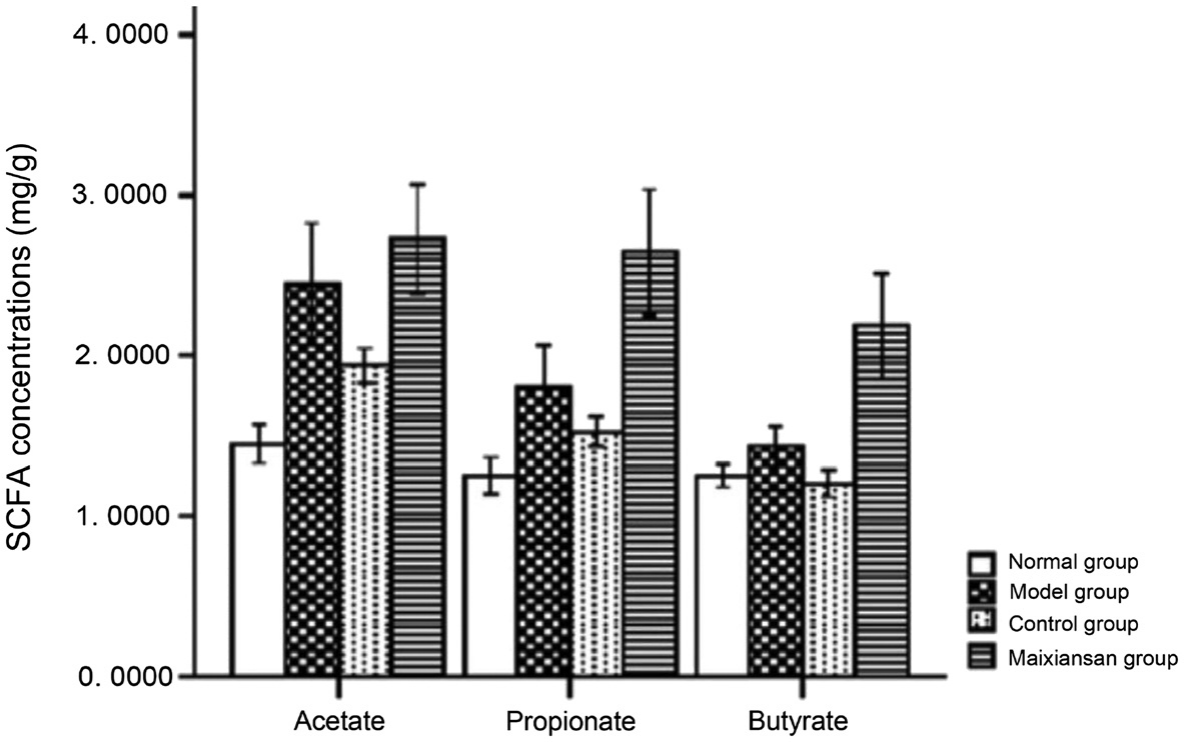
SCFA concentrations in rat stools from the different groups.

As shown in Figure 4, the SCFA contents in the Maixiansan-treated group contained significantly higher amounts of butyrate than the other groups. The mean butyrate concentration (mean ± SD of 10 rats) was 2.1847±0.4571 mg/g and a significant difference was observed between the Maixiansan-treated group and the control group (*P*=0.0035). Maixiansan composed of GBF passes through the intestines without being fully digested, and is fermented by the bacteria in the colon to release SCFAs. Butyrate is one of the SCFAs that can be used as a fuel by the cells lining the colon. SCFAs, particularly butyrate, are the major energy source for colonocytes [1,20]. Butyrate also has anti-inflammatory effects in certain states of mucosal inflammation and affects mucosal cell proliferation [21-23].

## Conclusion

Our experiments showed that RSM can optimize the extraction of DFM. Maixiansan could be explored as an anti-ulcerative colitis agent.

## Abbreviations

UC: Ulcerative colitis; SCFAs: Short-chain fatty acids; GBF: Germinated barley foodstuff; DFM: Dietary fiber from Maixiansan; RSM: Response surface methodology; BBD: Box–Behnken design; DSS: Dextran sodium sulfate; SD: Standard deviation; ANOVA: Analysis of variance.

## Competing interests

The authors declare that they have no competing interests.

## Authors’ contributions

HZ and CW designed the study. HL, CH and HZ performed the experiments, and wrote the manuscript. All authors read and approved the final manuscript.
